# Identification and validation of key genes associated with cell senescence in acute myocardial infarction

**DOI:** 10.3389/fcvm.2025.1499157

**Published:** 2025-02-19

**Authors:** Weidong Zhao, Guofu Zhu, Tianshu Chu, Liyong Wu, Hui Li, Qingwen Zhen, Rigui Wang

**Affiliations:** ^1^ Department of Cardiology, The Second Affiliated Hospital of Kunming Medical University, Kunming, Yunnan, China; ^2^ Department of Psychiatry, Yunnan Provincial Hospital of Infectious Diseases, Yunnan AIDS Care Center/Yunnan Mental Health Center, Anning, Yunnan, China

**Keywords:** acute myocardial infarction, cellular senescence, key genes, GEO, bioInformatic

## Abstract

**Introduction:**

Cellular senescence can cause heart failure. However, studies on diagnostic markers related to cellular senescence in acute myocardial infarction (AMI) have not been reported. Therefore, this study explores the mechanism of key genes related to cellular senescence in acute myocardial infarction (AMI) through a bioinformatics approach.

**Methods:**

AMI related datasets were obtained from gene expression omnibus (GEO) database, and 3,058 cellular senescence related genes (CSRGs) were extracted from Molecular Signatures Database (MSigDB). First, differentially expressed genes (DEGs) were screened by differential expression analysis,and then Weighted gene co—expression network analysis (WGCNA) was performed to obtained the key module. CSRGs, DEGs and genes in key module were intersected to acquire intersected genes, and candidate genes were also screened out by constructing a protein protein interaction (PPI) network.Afterwards, candidate genes were then subjected to a machine learning approach to identify key genes and enrichment analyses were performed on individual genes Finally, immuno-infiltrative analysis, competing-endogenous RNA (ceRNA) and drug-gene networks construction were conducted. Besides, the expression of key genes were validated by quantitative real-time polymerase chain reaction (qRT-PCR).

**Results:**

Screening for two key genes (ATP6V0B and DYNLL1) from 914 DEGs, and they were involved in functional pathways such as ‘mast cell activation’, ‘cytosolic ribosome’. Thereafter, we found infiltration of neutrophils, CD4 T cells memory resting and T cells gamma delta was notably different between AMI and control samples. Pearson correlation analysis suggested that the neutrophils had highest positive correlation with ATP6V0B (Cor = 0.7), while had highest negative correlation with DYNLL1 (Cor = −0.6). The ceRNA network had one mRNA (DYNLL1), one miRNA (hsa-miR-330-5p) and four circRNAs. Eventually, drug—ATP6V0B network had 74 nodes and 73 edges, drug—DYNLL1 network had 119 nodes and 118 edges. qRT-PCR suggested that the expression trend of DYNLL1 was consistent with the result of bioinformatic analysis. Notably, DYNLL1 was significantly down-regulated in the case group.

**Conclusion:**

Identified and validated DYNLL1 as a key gene related to cellular senescence in AMI, which is of great significance for the diagnosis and molecular targeted therapy of AMI.

## 1 Introduction

As we all know, the incidence, disability and mortality of cardiovascular disease (CVD) have increased dramatically, and acute myocardial infarction (AMI) plays a major role in CVD (1). In the past, we generally believed that AMI mainly occurred in middle-aged and elderly people, but with the development of society and the change of lifestyle, the incidence of AMI gradually showed a trend of younger and increasing year by year (2). AMI is caused by partial or complete coronary artery occlusion and results in cardiomyocytes ischemic injury, once cardiomyocytes are damaged by ischemia, the heart will be further fibrosis and remodeling, and eventually develop into heart failure (HF) or even sudden cardiac death (SCD) (3). With the development of medical technology, great progress have been made in the diagnosis and treatment strategies of AMI. At present, reperfusion therapy is the most effective therapeutic strategy, but many survivors still suffer from HF. The effective therapeutic targets to prevent cardiomyocytes remodeling are still limited, and further exploration of the pathogenesis for AMI at molecular level is needed (4). In recent years, although a lot of molecules and signaling pathways have been identified as potential therapeutic targets, new therapeutic strategies of promote cardiac repair after AMI have yet to be realized. Therefore, it is still a long way to explore the pathogenesis and to seek a more perfect molecular treatment strategy for AMI.

Cellular senescence is an irreversible cell cycle arrest induced by stress factors such as telomere dysfunction, DNA damage, chemotherapeutic drug damage, radiation damage, and oxidative damage, including replicative senescence and stress senescence (5–7). Senescent cells irreversibly lose the ability to proliferate and show flattened and enlarged in morphology (8). Senescent cells have an important characteristic: senescence associated secretory phenotype (SASP), which involves a series of inflammatory factors and has paracrine and autocrine effects on cells and tissues (7). Without specific markers, it is difficult to fully understand the biological process of cellular senescence. Senescence itself is a normal physiological process, however, it is associated with some undesirable aspects. It has been suggested that cellular senescence, accumulation of senescent cells, and production of SASP components are associated with cardiac diseases, such as HF, AMI, and cardiotoxicity associated with cancer chemotherapy (5). In addition, studies have also shown that cellular senescence is involved in cardiac regeneration, cardiac remodeling and HF after MI (9). Different types of cellular senescence in the heart can induce CVDs, such as atherosclerosis, MI, and cardiac fibrosis (6). The occurrence and development of cellular senescence in the heart is closely related to the pathophysiological process and severity of cardiac diseases (6, 10). Unfortunately, the exact role of senescent cells in cardiac disease is not clear. Some studies believe that transient cellular senescence in the heart may play a positive role, but long-term cellular senescence plays the opposite role (5, 9). Although numerous studies have explored the role of cellular senescence in cardiac diseases, there are still many unanswered questions. For example, in AMI, the potential benefits of targeted treatment for cellular senescence still unclear. Therefore, it is of great significance to further explore the role of cellular senescence in AMI.

In this study, the AMI dataset (GSE60993 and GSE66360) was downloaded from the Gene Expression Omnibus (GEO, http://www.ncbi.nlm.nih.gov/geo/) to explore potential key genes. Various bioinformatics analyses, including differential expression analysis, weighted gene co-expression network analysis (WGCNA), protein-protein interaction (PPI), and machine learning, were employed to identify key genes associated with AMI, immune cells, signaling pathways, regulatory networks, and potential therapeutic drugs. Overall, this study provide new insights into molecular targeted therapy and potential molecular mechanisms for AMI.

## 2 Materials and methods

### 2.1 Sources of data

The Gene Expression Omnibus (GEO)(*https://www.ncbi.nlm.nih.gov/gds*) database was used to obtain the training set GSE60993(17 acute myocardial infarction (AMI) samples and 7 control samples, samples type: human blood (Supplementary Table S1) and the validation set GSE66360(49 AMI samples and 50 control samples, samples type: circulating endothelial cells (Supplementary Table S2). Their detection platforms were *‘*Illumina HumanWG-6 v3.0 expression beadchip’ and ‘[HG-U133_Plus_2] Affymetrix Human Genome U133 Plus 2.0 Array’, respectively. Next, we extracted 3,058 cellular senescence related genes (CSRGs) via Molecular Signatures Database (MSigDB) (*https://www.gsea-msigdb.org/gsea/msigdb/index.jsp*) (with “cell aging AND Homo sapiens” as the keyword).

### 2.2 Differential expression analysis and WGCNA

Differentially expressed genes (DEGs) were identified by setting parameters of |log2FC| > 0.5 and *P* < 0.05 between AMI and normal samples in the GSE60993 dataset using the limma package (version 3.52.4) (11). In GSE60993 dataset, the co-expression network was constructed using the WGCNA package to identify gene modules most correlated with the traits of sample grouping (control and AMI). To ensure that the maximum mutual interaction between genes conforms to scale-free distribution, the determination of the soft threshold (β) for the data was conducted. Subsequently, based on the optimal soft threshold, each gene module was identified by setting the minimum number of genes to 100 according to the standards of the dynamic tree cutting algorithm. Correlations between genetic modules and traits were also examined, and the module found to correlate most strongly with AMI was identified as the principal module.

### 2.3 Functional enrichment and PPI analyses

The DEGs, CSRGs and genes within the key module were crossed to obtain common genes, which were analysed for GO (adj.*P* < 0.05) and kyoto encyclopedia of genes and genomes (KEGG) (*P* < 0.05) enrichment using the clusterProfiler package (version 4.4.4) (12). Moreover, in order to explore whether there were reciprocal relationships among intersected genes, we designed a PPI network via STRING database (*http://string-db.org*), and the genes with reciprocal relationships were screened out as candidate genes.

### 2.4 Screening for key genes by machine learning methods

In order to further screen out key genes that could be used as diagnostic markers for AMI, we applied machine learning algorithm analyses. First, least absolute shrinkage and selection operator (LASSO) logistic regression algorithm was converted to candidate genes using the glmnet package (version 4.1–4) (13), and gene coefficient and cross-validation error maps were gained. The importance degrees of candidate genes were ranked by support vector machine-recursive feature elimination (SVM-RFE) algorithmic, each iteration combination gives the error and accuracy rates, we then sifted out the genes with the lowest error rate point. Subsequently, key genes were identified by intersecting genes obtained through LASSO and SVM-RFE. To assess the discriminatory power of crucial genetic markers in identifying AMI from control samples, we generated a ROC curve using the pROC package (version 1.18.0) in the GSE60993 training dataset (14). In addition, in the GSE66360 validation set, we validated the scoring results using the same method. The ggplot2 package (version 3.3.6) (15) was employed to analyze gene expression differences between AMI and control samples in Tutorial Set GSE60993 and Verification Set GSE66360. Eventually, single gene GO and KEGG enrichment analyses of key genes were applied by clusterProfiler (version 4.4.4) and org.Hs.eg.db (version 3. 15.0) packages (12), setting |NES| > 1, NOM *P* < 0.05 and q < 0.25 (background gene sets: GO: c5.go.v2022.1.Hs.entrez.gmt, KEGG: c2.cp.kegg.v2022.1.Hs.en*trez.gmt*).

### 2.5 Immuno-infiltrative analysis

Above all, the distribution ratio of 22 immune cells was calculated for each sample in the GSE60993 training set was calculated, and immune cells with non-zero distribution ratio were extracted for correlation analysis. After that, principal component analysis (PCA) was implemented on AMI and control samples for immune cell infiltration. We compared cell infiltration differences between AMI and control samples using t-test. The association of key genes with immune cells was further investigated further with the help of Pearson's correlative degree analysis.

### 2.6 Construction of the competing endogenous RNA (ceRNA) and drug-gene networks

In the GSE160717 (3 AMI samples and 3 control samples, the testing platform was 074301 Arraystar Human CircRNA microarray V2) and GSE31568 (20 AMI samples and 64 control samples, the testing platform was ebit Homo Sapiens miRBase 13.0) datasets, we respectively screened out differentially expressed circRNAs (DE-circRNAs) and differentially expressed miRNAs (DE-miRNAs) by limma package (version 3.52.4) (11) setting *P* < 0.05 and |log2FC| > 0.5. Subsequently, the upstream miRNAs of key genes were predicted via miRNA target prediction and functional annotations database (miRDB) (*https://mirdb.org/mirdb/index.html*), and intersection with DE-miRNAs yielded the intersected miRNAs. CircRNAs targeting intersected miRNAs were forecasted by starbase database (*https://starbase.sysu.edu.cn/starbase2/*), and they were intersected with DE-circRNAs to yield intersected circRNAs. The relationship pairs of miRNA-mRNA and miRNA-circRNA with opposite trend of expression were retained. Consequently, we constructed a ceRNA network based on the above results. Furthermore, we utilized comparative toxicogenomics database (CTD) database to predict the target therapeutic drugs and molecular compounds of key genes, and constructed drug (molecular compound)-gene networks.

### 2.7 Quantitative real-time polymerase chain reaction (qRT-PCR)

The blood samples were separated using PBMC separation solution, and total RNA was eluted using with TRIzol reagents. The RNA concentration was measured using NanoPhotometer N50 after using 1 ul of the sample. Surescript-primer-cDNA-synthe-kit from Servicebio was usilized to perform reverse transcription of the mRNA. The resulting cDNA was amplified 5–20 times with RNase/DNase-free ddH2O before being subjected to qPCR reaction. The gene detection used GAPDH as the internal reference. A comparison of key gene expressions between the case and normal groups was performed. Sequences can be found in Table 1.

**Table 1:** The primer sequences of key genes.

Primer	Sequence
ATP6V0B F	GATTTGGGCTTCCGCTTTGAT
ATP6V0B R	TGCCATGATGATGCCGTAGAT
DYNLL1 F	AGAAGGACATTGCGGCTCAT
DYNLL1 R	GCCACTTGGCCCAGGTAGAA
IC-GAPDH F	CGAAGGTGGAGTCAACGGATTT
IC-GAPDH R	ATGGGTGGAATCATATTGGAAC

This study was approved by the Ethics Committee of the Second Affiliated Hospital of Kunming Medical University. Informed consent was obtained from all subjects involved in the study.(Audit-PJ-Science-2023-139, 2023-7-12). We collected fresh blood samples of case (*n* = 10) and normal (*n* = 10) groups from Cardiovascular Department, the Second Affiliated Hospital of Kunming Medical University. The 10 patients with AMI included 6 males and 4 females, including 7 STEMI and 3 NSTEMI. The normal control group (5 men and 5 women) were healthy volunteers who went to the hospital for health examination at the same time, without any history of CVD. According to the guidelines adopted by the American Heart Association/American Heart Association (2018 ESC/ACC/AHA/WHF Fourth General Definition), all patients had a first-time diagnosis of AMI and had been assessed by two independent cardiologists. After being diagnosed with AMI and obtaining consent, 5 ml of venous blood was collected immediately and PBMC isolation was performed. The exclusion criteria for this study were severe liver and kidney failure, severe infectious disease, autoimmune disease, malignancy, hematological disease, and previous history of cardiovascular disease (Table 2).

**Table 2:** The basic information.

Group	Sex		Age	BMI	Smoking (*n* %)	Hypertension (*n* %)	T2DM (*n* %)	COPD (*n* %)	Hypercholesterolemia (*n* %)	Family genetic history (*n* %)
	Male (*n* %)	Female (*n* %)								
AMI (*n* = 10)	6 (60%)	4 (40%)	71.4 ± 8.83	24.58 ± 1.71	5 (50%)	6 (60%)	3 (30%)	2 (20%)	5 (50%)	4 (40%)
Control (*n* = 10)	5 (50%)	5 (50%)	69.50 ± 9.17	23.53 ± 1.08	3 (30%)	5 (50%)	2 (20%)	2 (20%)	3 (30%)	4 (40%)
P	0.653	\	0.643	0.118	0.361	0.653	0.606	1.000	0.361	1.000

## 3 Results

### 3.1 A total of 914 DEGs and 1, 076 key module were identified in GSE60993 dataset

**Figure 1:**
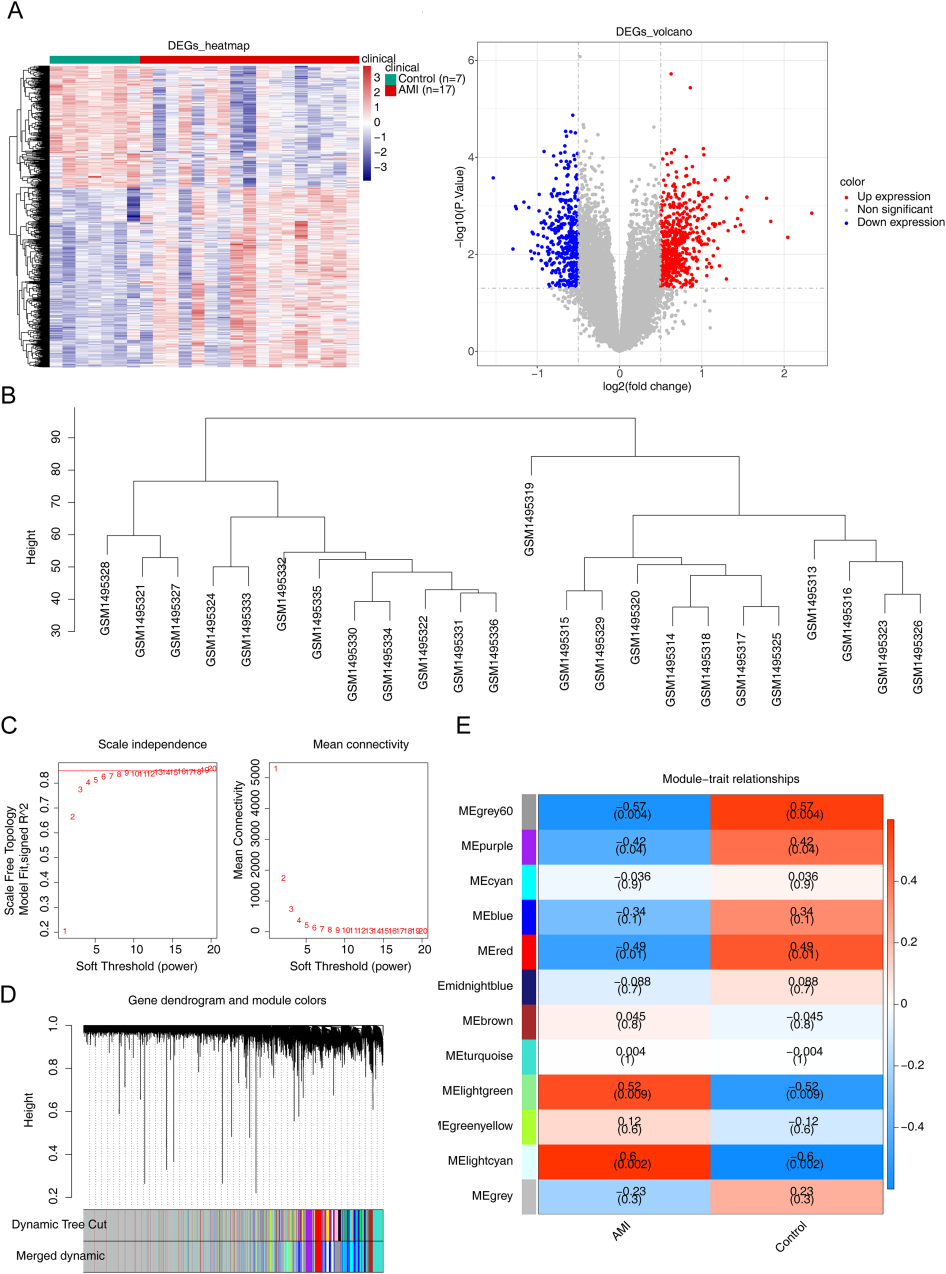
Differential expression analysis in the GSE60993 dataset. **(A)** The heatmap and volcano map of differentially expressed genes (DEGs) between acute myocardial infarction (AMI) and control samples. **(B)** The samples were clustered to remove the outlier. **(C)** Selection of the optimal soft-threshold (power). **(D)** Hierarchical clustering of genes and module identification. **(E)** Heatmap of the relationships between gene modules and clinical traits (AMI and control).

In the GSE60993 dataset, a total of 914 DEGs were identified across AMI and control specimens, as shown in Figure 1A; Supplementary Table S3. Figure 1B demonstrated that the dataset samples were well-clustered, thereby indicating no need to exclude any samples. The power limit was established at 19 based on the placement of the red line in Figure 1C. At this point, the network had approached the scaleless delivery with a stagnant tendency, because the perpendicular scale *R*^2^ was about 0.85, and the average value of the proximity curve was progressive approaching 0. We constructed the co-expression matrix (Figure 1D) to sift out 12 modules, which included merged modules. Ultimately, we discovered that the lightcyan module (containing 1,076 genes) exhibited the strongest correlation with AMI (*R* = 0.6, *P* = 0.002), thus it was identified as the principal module (Figure 1E).

### 3.2 42 intersected genes were enriched in various GO items and KEGG pathways

**Figure 2:**
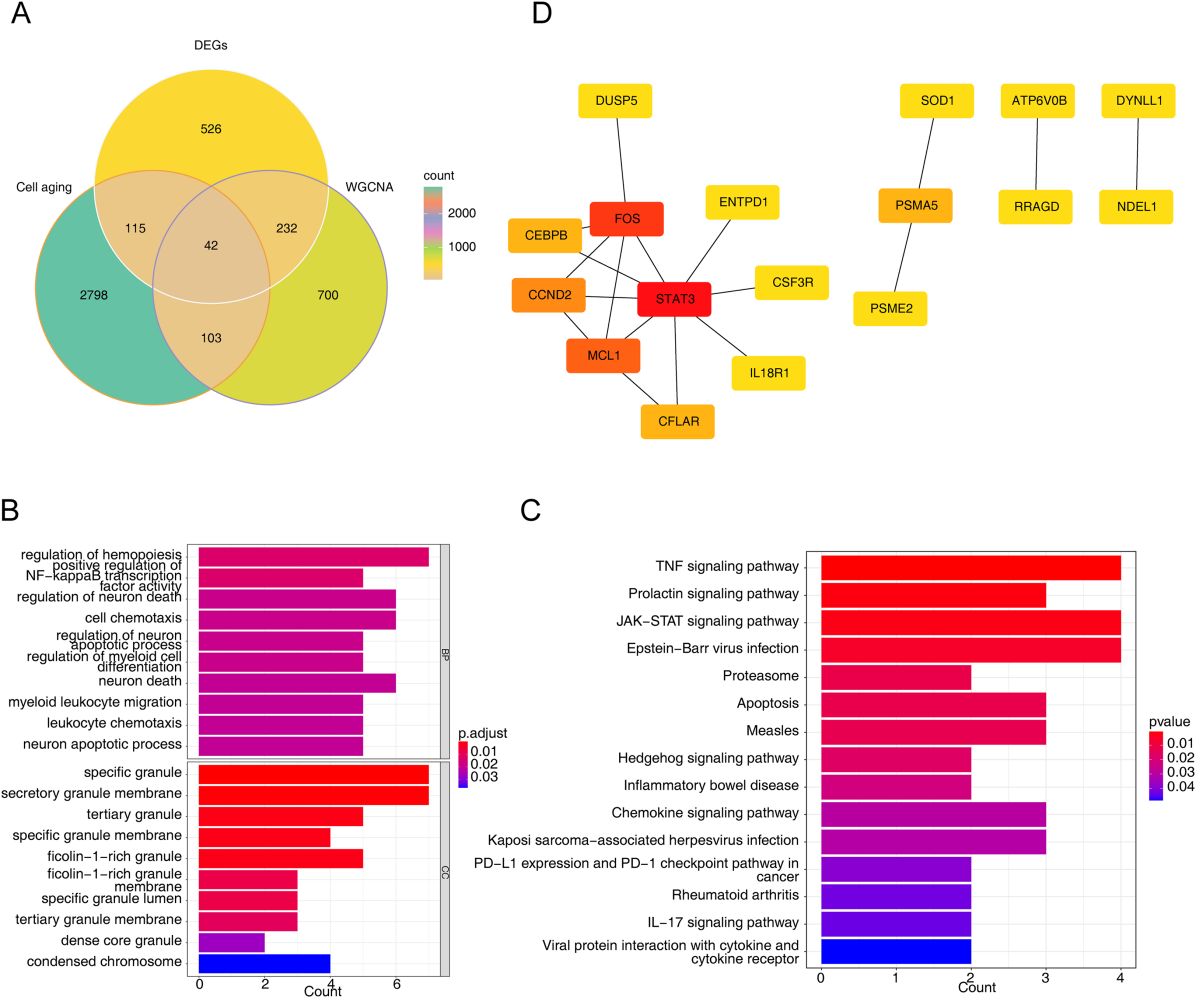
Identification of intersected genes and functional enrichment analysis. **(A)** The venn diagram of 42 intersected genes obtained by overlapping cellular senescence related genes (CSRGs), DEGs, and module key module genes in weighted gene co-expression network analysis (WGCNA). **(B,C)** The gene ontology (GO) terms **(B)** and Kyoto encyclopedia of genes and genomes (KEGG) pathways **(C)** enriched in 42 intersected genes. BP, biological progress; CC, cellular component. **(D)** The protein-protein interaction (PPI) network of intersected genes.

The intersection of CSRGs, DEGs and genes in key module resulted in 42 intersected genes (Figure 2A). Besides, overlapping genes were linked to GO annotation records such as ‘regulation of hemopoiesis’, ‘specific granule’, ‘regulation of neuron apoptotic process’, *‘*neuron death’, ‘condensed chromosome’ (Figure 2B). Meanwhile, they were enriched to KEGG pathways such as ‘apoptosis’, *‘*chemokine signaling pathway’, ‘IL-17 signaling pathway’, ‘TNF signaling pathway’, and ‘JAK-STAT signaling pathway’ (Figure 2C). Eventually, 17 nodes (candidate genes) and 18 edges constituted a PPI network, including FOS-STAT3, MCL1-CCND2, PSMA5-PSME2 and other reciprocal relationship pairs (Figure 2D).

### 3.3 ATP6V0B and DYNLL1 were screened as key genes

**Figure 3:**
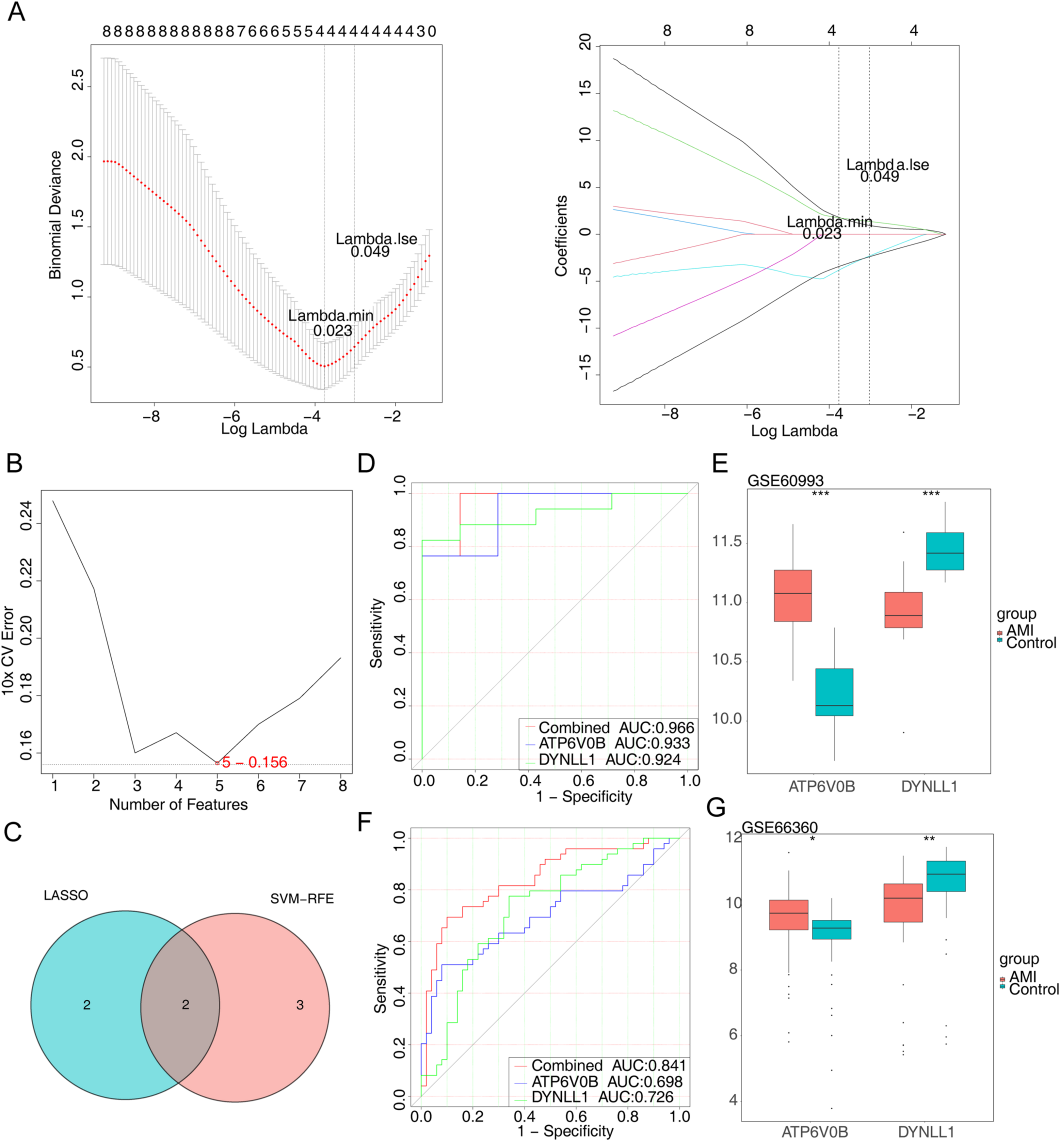
Identification of key genes. **(A)** The least absolute shrinkage and selection operator (LASSO) coefficient distribution of four candidate genes, different colors represent different genes. **(B)** The results of support vector machine-recursive feature elimination (SVM-RFE) algorithm. **(C)** The venn diagram of two key genes. **(D)** The receiver operating characteristic (ROC) curves of key genes. AUC, area under the curve. **(E)** The expression of key genes in AMI and control samples. **(F)** The ROC curves of key genes in the GSE66360 dataset. **(G)** The expression of key genes in the GSE66360 dataset.

When lambda = 0.023, error rate was the lowest, and we obtained four genes (ATP6V0B, CEBPB, DYNLL1 and PSME2) at this point (Figure 3A). We also yielded five genes (IL18R1, RRAGD, DYNLL1, ATP6V0B and FOS) via SVM-RFE (Figure 3B). The two key genes, namely ATP6V0B and DYNLL1, were identified after taking the crossing genes obtained by LASSO and SVM-RFE (Figure 3C). Besides, area under the curve (AUC) values of ATP6V0B (AUC = 0.933) and DYNLL1 (AUC = 0.924) both exceeded 0.9, indicating that they had excellent ability to distinguish AMI from control samples (Figure 3D). Finally, we found the expressions of ATP6V0B and DYNLL1 were all notably different between AMI and control samples. Specifically, ATP6V0B was significantly overexpressed in the AMI group, while DYNLL1 was significantly under expressed in the same group (Figure 3E). The diagnostic ability and expression of key genes were further validated in the GSE66360 validation set (Figures 3F,G).

### 3.4 Both ATP6V0B and DYNLL1 were enriched in ribosome-related function

**Figure 4:**
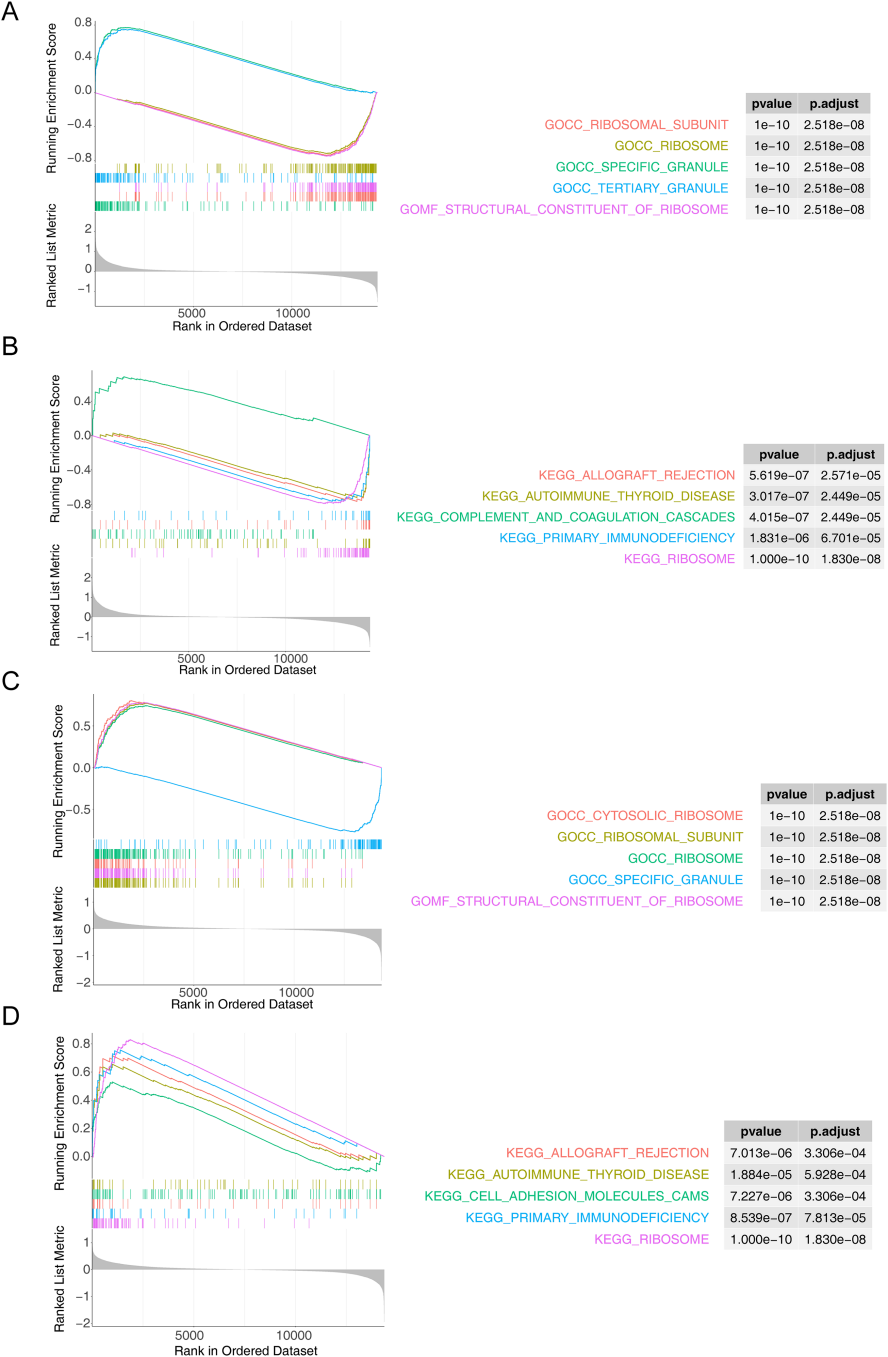
Functional enrichment analysis. **(A,B)** The GO term **(A)** and KEGG **(B)** pathways enriched in ATP6V0B. **(C,D)** The results of GO **(C)** and KEGG **(D)** enrichment analysis for DYNLL1.

ATP6V0B was enriched to 547 GO entries and 40 KEGG pathways, of which GO entries included *‘*mast cell activation’, ‘ribosomal subunit’, ‘specific granule’, ‘tertiary granule’, ‘gamma delta *T* cell activation’ and others (Figure 4A), KEGG pathways contained ‘ribosome’, ‘complement and coagulation cascades’, ‘primary immunodeficiency’, ‘Toll like receptor signaling pathway’, ‘DNA replication’ and others (Figure 4B). Moreover, DYNLL1 was associated with 429 GO entries and 38 KEGG pathways, such as ‘cytosolic ribosome (GO)’, ‘cytoplasmic translation (GO)’, ‘mitochondrial protein containing complex (GO)’, ‘mitochondrial gene expression (GO)’, ‘ncRNA processing (GO)’, ‘allograft rejection (KEGG)’, ‘cell adhesion molecules cams (KEGG)’, ‘FC gamma R mediated phagocytosis (KEGG)’, *‘*nucleotide excision repair (KEGG)’, ‘purine metabolism (KEGG)’, etc. (Figures 4C,D).

### 3.5 Immune microenvironment analysis

**Figure 5:**
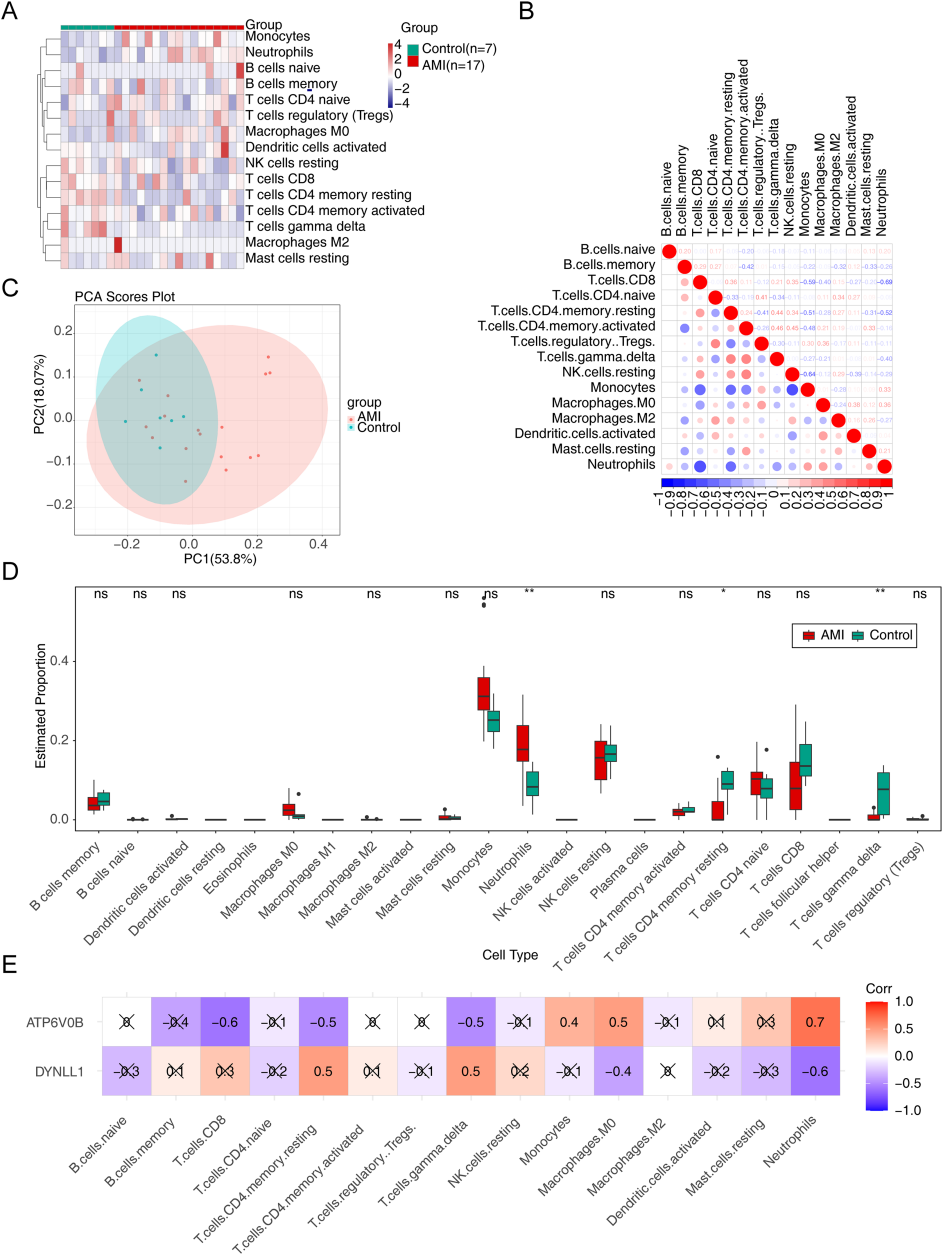
Immune infiltration analysis. **(A)** The infiltration of immune cells in AMI and control samples. **(B)** The relevance of immune cells. **(C)** The principal component analysis (PCA) of AMI and control samples. **(D)** The discrepancies of immune cells in AMI and control samples. ns, not significant; *p < 0.05; **p < 0.01. **(E)** The relevance of key genes to immune cells.

The heatmap displayed the infiltration proportions of immune cells in all samples of the GSE60993 dataset, with 15 immune cell types having non-zero distribution ratios, thus being included in subsequent analysis (Figure 5A). Meanwhile, of these 15 immune cells, monocytes and neutrophils had the strong negative correlations with other immune cells (Figure 5B). PCA showed that the clustering between AMI and control samples was excellent (Figure 5C). Thereafter, significant differences in the infiltration of neutrophils, resting memory CD4 *T* cells, and gamma delta *T* cells were found between AMI and control samples, with a significantly higher proportion of neutrophils and lower proportions of resting memory CD4T cells and gamma delta *T* cells in the AMI group. (Figure 5D*).* Pearson correlation analysis suggested that neutrophils exhibited the highest positive correlation with ATP6V0B (Cor = 0.7), while had the highest negative correlation with DYNLL1 (Cor = −0.6) (Figure 5E).

### 3.6 The construction of circRNA-miRNA-mRNA and drug-key gene networks

**Figure 6:**
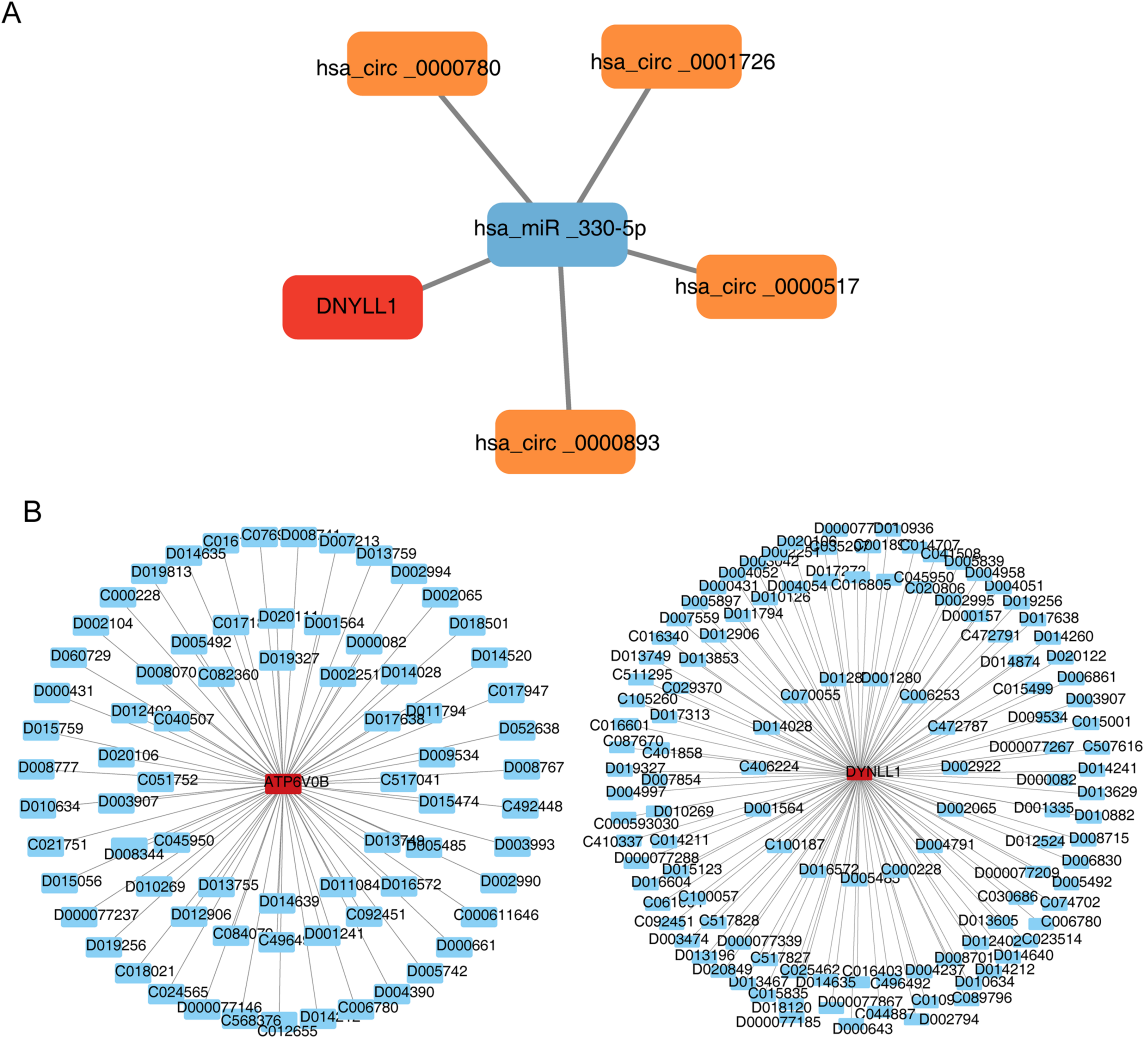
Construction of regulation network. **(A)** The competing endogenous RNA (ceRNA) network of DYNLL1. The red graphic represents gene, blue represents microRNA (miRNA), and orange represents circular RNA (circRNA). **(B)** The gene-drug network of key genes. The red graphic represents genes and blue represents drug.

The ceRNA network consisted of one mRNA (DYNLL1), one miRNA (hsa-miR-330-5p) and four circRNAs (hsa-circ-0000780, hsa-circ-0001726, hsa-circ-0000517 and hsa-circ-0000893), including‘hsa-circ-0000780’-‘hsa-miR-330-5p’-DYNLL1, *‘*hsa-circ-0001726’-‘hsa-miR-330-5p’-DYNLL1,‘hsa-circ-0000517’-‘hsa-miR-330-5p’-DYNLL1 and other relationship pairs (Figure 6A). Additionally, we found that cyclosporine and decamethrin could increase the expression of ATP6V0B, while acrylamide enhances the production of DYNLL1, aconitine reduces it. Figure 6B demonstrated the drug-key gene networks.

### 3.7 Significant downregulation of DYNLL1 in AMI groups in results of qRT-PCR

**Figure 7:**
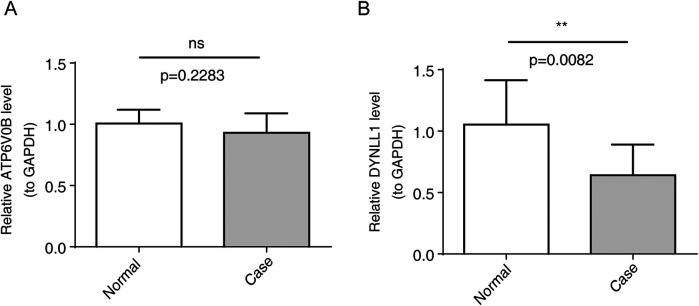
The expression of key genes by qRT-PCR. **(A)** Relative ATP6V0B level, ns, not significant. **(B)** Relative DNYLL1 level, **p < 0.01.

To further validate the expression of key genes, blood samples from 10 AMI and 10 control samples were collected for qRT-PCR. DYNLL1 was found to be significantly downregulated in the AMI groups (Figure 7), and its expression trend was consistent with the results of bioinformatic analysis. However, no significant difference in ATP6V0B expression was observed between AMI and control samples.

## 4 Discussion

According to statistics, AMI accounts for 20% of deaths worldwide (16). Previous studies have shown that cardiac troponin is the most clinically significant diagnostic index for AMI (17). However, the prediction efficiency and specificity of cardiac troponin are still poor. Therefore, it is of great significance to search for new diagnostic biomarkers. In addition, the current treatment methods still cannot fundamentally solve the consequences of AMI, and many patients still suffer from HF, arrhythmia, and even SCD after AMI. Therefore, we need to explore more perfect molecular targeted therapy strategies.

Cellular senescence occurs in proliferating cells and suffers from various stress factors, which manifest as morphological changes, altered gene expression, and the secretion of large amounts of cytokines (7). Senescence has been shown to have a dual role in cancer (18), however, the positive or negative role in the heart has not been fully elucidated. During cardiac cell senescence, cell stiffness, fibrosis and apoptosis increase, thereby promoting HF (10). A study found that senescent foam cells are detrimental in the process of atherosclerosis, and that cellular senescence as key point for the formation and maturation of atherosclerosis, and that remove these senescent cells is expected to treat atherosclerosis (19). In cancer, therapies that modulate the senescence responses are in the pre-clinical or clinical stages currently (18). Unfortunately, these therapeutic strategies are still uncharted territory in heart disease.

Most bioinformatics analyses aimed to find key genes for AMI, even studies identified key genes associated with aging in COPD and idiopathic pulmonary fibrosis (20, 21). However, our study aimed to find key genes associated with cellular senescence in AMI. Xiang et al. identified three key genes related to senescence in endothelial cells from patients with AMI: MMP9, ETS2, and BCL6 (22). In this study, we identified two key genes related to cellular senescence in patients with AMI: ATP6V0B and DYNLL1. We also found that DYLL1 was significantly down-regulated in the case group, and its expression trend was consistent with the results of bioinformatics analysis, through qRT-PCR. Finally, we validated and identified DYNLL1 as a key gene related to cellular senescence in AMI.

In cardiovascular related research, gender and age differences may have an impact on gene expression. In terms of gender, sex hormones play different physiological roles in the cardiovascular system of males and females, Sex-related and gender-related factors often have opposite effects on the clinical manifestations and outcomes of cardiovascular disease (23). In terms of age, the incidence rate of AMI increases with age, and age itself may worsen the outcome after AMI (24). When AMI occurs, young patients may have a more active and effective immune response to clear necrotic tissue and initiate repair processes, while elderly patients may experience excessive inflammatory response or insufficient repair (25).Therefore, the expression of key genes related to the pathophysiological process of AMI may be different depending on age and sex.

DYNLL1, also known as dynein light chain LC8-1(DLC1, LC8) is the smallest and highly conserved member of the dynein family, it is a multifunctional protein and a hub protein, which proposed to regulate mitochondrial apoptosis (26). However, apoptosis is an important pathological change after AMI. Therefore, we can speculate that there is a correlation between DYNLL1 and AMI. Estellen et al. showed that LC8 is locate at the center of a complex interaction network and is essential for cell homeostasis as a hub protein (27). According to the swift et al’ study, DYNLL1 regulates 53BP1 and interact with chromatin associated with DNA double-strand break (DSB), regulates 53BP1-dependent non-homologous end joining (NHEJ) (28). Zalmas et al. showed that DYNLL1 regulates the localization and function of 53BP1, which may be involved in telomere end protection (29). Swift et al. found that DYNLL1 is an anti-excision factor, which can significantly affects genomic stability and the response to DNA chemotherapy damage (30). Most studies on DYNLL1 have focused on cancer. For example, Berkel C et al. found that DYNLL1 may play an important role in ovarian cancer progression and chemical resistance (31).

It is worth noting that the role of DYNLL1 in AMI and cellular senescence belongs to an entirely new field. In our study, the expression of DYNLL1 was significantly reduced in patients with AMI, while ATP6V0B was the opposite. They were evaluated and validated by ROC curve and external validation set. The results show that ATP6V0B and DYNLL1 have good diagnostic efficiency for AMI both in vascular epidermal cells and blood samples, which provides new insights for the recognition and treatment of AMI. Unfortunately, ATP6V0B as one of the four subunits of the lysosomal soma pump (32), was found not statistically significant by qRT-PCR in this study.

In our study, we also performed single-gene GO and KEGG enrichment analyses for these two key genes to further explore their signaling pathways and potential biological mechanisms. DYNLL1 was associated with 429 GO items and 38 KEGG pathways, among which the top 5 GO enriched items included cellular ribosome, cytoplasmic translation, mitochondrial protein containing complex, mitochondrial gene expression, ncRNA processing. KEGG analysis showed that DYNLL1 was mainly related to allograft rejection, cell adhesion molecules, FcγR-mediated phagocytosis, nucleotide excision repair, purine metabolism, etc. Marinescu et al. show that three types of ncRNA (miRNA, lncRNA, and circRNA) as signaling molecules closely related to CVD, were involved in the signaling pathway of cardiomyocyte death and cardiomyocyte regeneration, and that novel strategies targeting ncRNA may be beneficial to AMI and improve clinical outcomes (33). Aydin et al. found that cell adhesion molecules (CAMs), as candidate biomarkers of AMI, may play a role in the diagnosis of AMI (17). Lino et al. found that plasma vascular cell adhesion molecule-1 (VCAM-1) and intercellular adhesion molecule-1 (ICAM-1) were increased in patients with HF after AMI, and that it may serve as biomarkers for HF after AMI and have a significant contribution to the prediction of HF after AMI (34). Meyer et al. found that cell senescence is associated with increased VCAM-1 and ICAM-1, and that endothelial senescence may lead to microvascular complications through the secretion of VCAM-1/ICAM-1 (35). Uric acid is a xanthine metabolite, which plays an antioxidant role. Many studies have confirmed that uric acid is associated with CVD.The other GO enrichment items and KEGG pathways have not been reported in relation to AMI and cellular senescence.

In our study, we also performed immune infiltration analysis and found that Neutrophils, CD4 *T* cells memory resting and γδ *T* cells were significantly different between AMI and controls, and that Neutrophils had the highest positive correlation with ATP6V0B and the highest negative correlation with DYNLL1. After AMI, cytokines released by necrotic cardiomyocytes can activate a strong inflammatory response, which in turn mediates scar formation to promote myocardial repair, but excessive inflammatory response may induce poor myocardial remodeling (fibrosis and scar) and then promote HF (36). A study published in Circulation showed that neutrophilia, as a core part of the inflammatory response, was related to MACE in patients with AMI (37). Kologrivova et al. showed that neutrophils play a major role in inflammation resolution and cardiac repair after AMI, and that elevated neutrophils are associated with poor prognosis in patients with AMI (38). It has also been shown that neutrophils induce telomere dysfunction and senescence in ROS-dependent manner, and that neutrophils are both recruited by senescent cells and can act as drivers of senescence (39), but this study used mouse liver as the specimen. In our study, the expression of DYNLL1 was significantly reduced in patients with AMI, and the neutrophils had highest negative correlation with DYNLL1, which is consistent with previous studies. It can also be speculated that there is a link between DYNLL1 and AMI. Similarly, many previous bioinformatics analyses have suggested that γδ *T* cells and CD4T cells memory resting may be involved in the development and progression of AMI (40, 41). These findings are consistent with those of our study, and which confirm the high accuracy of our study.

This study also constructed a ceRNA network, which consist of four regulatory axes: hsa circ-0000780/hsa miR-330-5p/DYNLL1, hsa circ-0001726/hsa miR-330-5p/DYNLL1, hsa circ-0000517/hsa miR-330-5p/DDYLL1, and hsa cir-0000893/hsa miR-330-5p/DDYLL1. According to previous studies, CircRNA is involved in apoptosis, fibrosis, inflammatory response, and cardiac repair after AMI, and may also serve as a diagnostic biomarker for AMI (42). Similarly, miRNAs are involved in various pathophysiological processes of AMI and may serve as a novel biomarker for the diagnosis and prognosis of AMI (43). Most studies have shown that CircRNAs mainly functions through binding to miRNA or RNA binding protein (RBP) and regulating parental gene transcription, and that participating in and regulating the pathophysiological process of AMI through different circRNA/miRNA/mRNA axes (44). Some CircRNAs are also involved in and play a role in aging and may be pathogenic factors in age-related diseases (45). The study by Niu et al. found that many CircRNAs are related to aging and longevity, positively and negatively regulating aging and longevity through typical aging pathways (46). Hsa-miR-330-5p, hsa-circ-0000780, hsa-circ-0001726, hsa-circ-0000517 and hsa-Circ-0000893 in the ceRNA regulatory network have not been reported to be related to AMI or aging. In the future, we will further investigate the relationship between the CircRNA/miRNA/mRNA axis and AMI.

Finally, this study also explored targeted drugs related to senescence. *Senotherapeutics* have been explored for eliminating senescent cells, known as senolytics, reducing the harmful effects of SASP or inhibiting aging compounds, known as senomorphics, also known as senostatics. These two targeted therapy strategies are already in clinical or preclinical research stages (7, 47). It has been shown that after ischemia-reperfusion injury, treatment with navitoclax can eliminate aging cells in the heart, reduce SASP mediated inflammation, increase myocardial vascularization, reduce scar size, and improve cardiac function (48). In our study, we found that cyclosporine and decamethrin could increase the expression of ATP6V0B, and that acrylamide (ACR) could increase the expression of DYNLL1 while aconitine had opposite effect. At present, there is a large amount of evidence for the carcinogenicity of ACR, and a few studies have also shown the toxic effects of ACR in cellular senescence. For example, Mahdizade et al. showed that ACR enhances the senescence response of mouse embryonic fibroblasts (49). It is also reported that 3% gelatin Methacrylamide can synthesize a gelatin based cell coating, which can improve the retention of Bone Marrow Derived Cell in heart tissue after MI, opening up a new way for the research of heart regeneration therapy (50). The cardiotoxicity and neurotoxicity of Aconitine (AC) have attracted much attention,but it has not been reported in AMI or Cellular senescence. Therefore, we need more research to explore the beneficial or harmful effects of Acrylamide and Aconitine on AMI.

In this study, the key genes related to cell senescence in AMI were identified by bioinformatics methods, and the enrichment analysis, immune infiltration analysis, potential drug analysis and ceRNA network construction were carried out for key genes. The blood sample in patients with AMI were collected for PCR verification, unfortunately, only one of the key genes was statistically significant. At present, the intervention measures to remove senescent cells and inhibit cellular senescence are mainly focus on cancer. This study provides the possibility for breakthroughs in the field of new diagnostic markers and molecular Targeted therapy of AMI.

However, this study also has limitations. First, the sample size is small. A small sample size may not be able to fully cover the various risk factors in patients with AMI, which to some extent limits the representativeness and accuracy of the findings. Second, the study did not take fully into account gender and age. In the study of AMI, the influence of gender and age on the disease cannot be ignored. There may be significant differences in physiological function, susceptibility to disease and gene expression among patients of different genders and ages. Because sex and age stratification were not performed, it is likely that we missed changes in gene expression that were significantly different in some specific sex or age group, thus affecting the accurate judgment of the study results. However, we have recognized the importance of gender and age factors in AMI research. Therefore, in future studies, we will aim to obtain a more complete data set that includes gender and age information to more fully explore the impact of these factors on the study results to improve the reliability and validity of the study.There are also many challenges, such as the lack of specific biomarkers in aging cells, making it difficult to recognize aging cells in the heart.

In future, we will continue to conduct in-depth research on the beneficial or harmful effects of DYNLL1 on AMI, the regulatory mechanism of DYNLL1 on AMI, the cardiac pathological changes after overexpression or knockout of DYNLL1, and new treatments strategies for DYNLL1 in AMI.

## Data Availability

The original contributions presented in the study are included in the article/Supplementary Material, further inquiries can be directed to the corresponding author.

## Supplementary material

Supplementary Table S1 Sample information in dataset GSE60993.

Supplementary Table S2 Sample information in dataset GSE66360.

Supplementary Table S3 The DEGs between AMI and control samples.
